# 

**Published:** 1891-07-11

**Authors:** 


					?aiHULnvv
AND INVALIDS.
for INFANTS
lift
HOWAS'S blOSP.
1RWICH haspl.TAJ.
.ASQoir Western IHriRMA&f
WITH -EXTRA 'NURSIKG SUPPi.EMENT.
No. 250. Vol. x.1
Saturday,
July 11th, 1891.
[One Pbnny.

				

